# Not all Bosniak category IV cysts are malignant: foreign body granuloma mimicking renal cell carcinoma

**DOI:** 10.1590/0100-3984.2017.0197

**Published:** 2019

**Authors:** Lucas de Pádua Gomes de Farias, Igor Gomes Padilha, Carla Jotta Justo dos Santos, Christiana Maia Nobre Rocha de Miranda

**Affiliations:** 1 Universidade Federal de Alagoas (UFAL), Maceió, AL, Brazil; 2 Clínica de Medicina Nuclear e Radiologia de Maceió (MedRadius), Maceió, AL, Brazil

**Figure 1:**
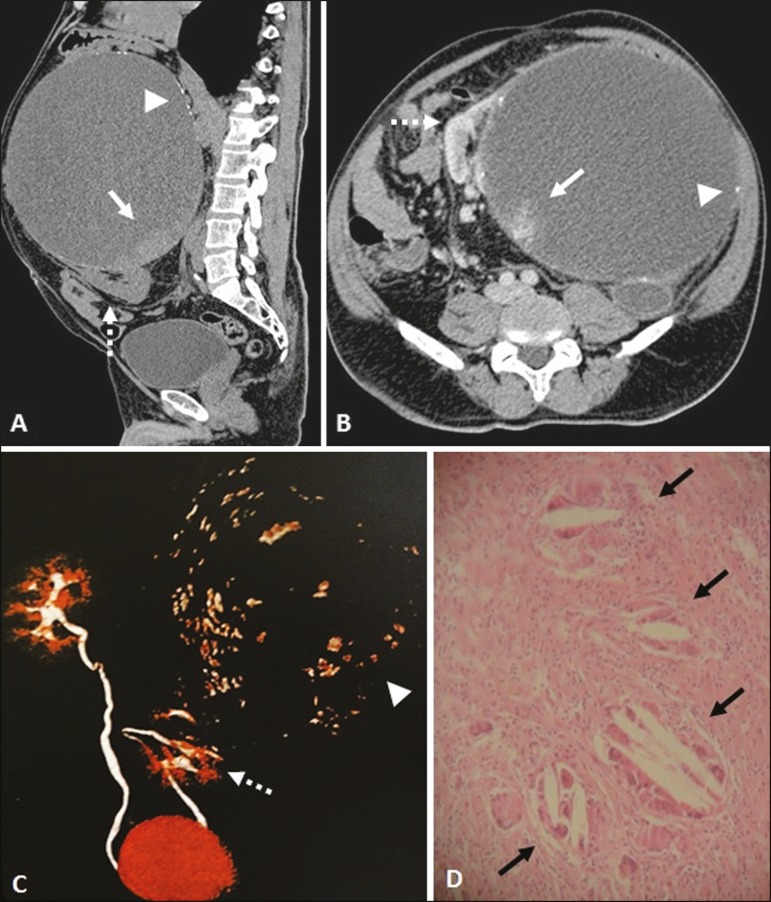
Multidetector computed tomography. Unenhanced sagittal slice (**A**), intravenous contrast-enhanced axial slice (**B**), and three-dimensional reconstruction (**C**), showing a voluminous cystic formation, classified as Bosniak category IV, in juxtaposition with the renal parenchyma (dashed arrows) in the left kidney, containing diffuse calcifications in its walls (arrowheads) and a solid component, with contrast enhancement in the inferior aspect of the cyst (arrows). **D:** Histological slide of the solid component with hematoxylin-eosin staining (magnification, 10×), showing foreign body reaction-like granulomatous inflammatory process (black arrow), with no evidence of malignancy.

Dear Editor,

A 62-year-old male patient presented with a five-year history of increased abdominal volume, without pain, discomfort, fever, weight loss, or any complaints related to the urinary or gastrointestinal tract. He reported no history of trauma or surgery. Multidetector computed tomography revealed a voluminous cyst in the left kidney (Figures 1A, 1B, and 1C). The cyst contained parietal calcifications and a solid component, showing contrast enhancement (Bosniak category IV), accompanied by atypical enlargement of the retroperitoneal lymph nodes and bilateral simple renal cysts. The patient underwent total nephrectomy, and the histopathological analysis of the excised kidney revealed a granulomatous foreign body reaction in the form of an inflammatory process, chronic pyelonephritis with glomerular hyalinization, and the absence of malignancy (Figure 1D).

Renal cysts are the most common findings in day-to-day radiology practice, and their diagnosis by imaging methods is simple and accurate. However, complex cysts and cysts with solid components require further characterization for the evaluation of the differential diagnoses and for treatment planning^(1-3)^. In 1986, Bosniak^(4)^ developed a classification system, based on computed tomography imaging criteria, that allows the analysis of aspects related to the contour and content of the renal cyst, the presence of septations or calcifications, and the degree of enhancement after intravenous administration of contrast media, ranking them in ascending order by the likelihood of malignancy^(1,2,4)^: category I (simple cyst); category II (minimally complicated cyst); category IIF (minimally complicated cyst, requiring follow-up); category III (cyst of undetermined nature, requiring excision); and category IV (cystic neoplasm, requiring excision).

Lesions classified as Bosniak category IV are cystic neoplasms with solid components that show contrast enhancement, adjacent to the lesion wall or accompanied by thickened or nodular septa, and can also present wall thickening. They are considered renal cell carcinomas (RCCs) until proven otherwise^(1,5)^. A foreign body granuloma is uncommon and is indistinguishable from an RCC, the most common solid renal tumor, because the two have similar radiological characteristics^(5)^.

The imaging characteristics of foreign body granuloma of the kidney are extremely varied, depending on the presence of calcification, an adipose component, and necrosis. Because of those characteristics, it is quite difficult to distinguish among foreign body granuloma, RCC, and angiomyolipoma on the basis of imaging findings. Other differential diagnoses include transitional cell carcinoma, oncocytoma, lymphoma, leiomyoma, xanthogranulomatous pyelonephritis, and Erdheim-Chester disease^(2,3,5-7)^.

With the increase in the number of laparoscopic partial nephrectomies, the incidence of foreign body granuloma also increased^(5)^. When an expansile renal lesion exhibits calcifications, contains adipose foci, is well encapsulated, and shows no signs of infiltration of adjacent structures, the possibility of granuloma should be considered, especially if there is a history of surgical manipulation of the urinary tract^(6)^. It is also noteworthy that renal granulomas are not exclusively associated with foreign bodies in the renal parenchyma. They can be due to systemic diseases, be caused by inert endogenous substances, or even have an idiopathic etiology^(8)^.
